# The Sun’s Vitamin in Adult Patients Affected by Prader–Willi Syndrome

**DOI:** 10.3390/nu12041132

**Published:** 2020-04-17

**Authors:** Luigi Barrea, Giovanna Muscogiuri, Gabriella Pugliese, Sara Aprano, Giulia de Alteriis, Carolina Di Somma, Annamaria Colao, Silvia Savastano

**Affiliations:** 1 Dipartimento di Medicina Clinica e Chirurgia, Unit of Endocrinology, Federico II University Medical School of Naples, Via Sergio Pansini 5, 80131 Naples, Italy; giovanna.muscogiuri@gmail.com (G.M.); robiniapugliese@gmail.com (G.P.); saraaprano@hotmail.com (S.A.); dealteriisgiulia@gmail.com (G.d.A.); cdisomma@unina.it (C.D.S.); colao@unina.it (A.C.); sisavast@unina.it (S.S.); 2 Centro Italiano per la cura e il Benessere del paziente con Obesità (C.I.B.O), Department of Clinical Medicine and Surgery, Endocrinology Unit, University Medical School of Naples, Via Sergio Pansini 5, 80131 Naples, Italy; 3 Cattedra Unesco “Educazione alla salute e allo sviluppo sostenibile”, University Federico II, 80138 Naples, Italy

**Keywords:** Prader–Willi syndrome (PWS), vitamin D, dietary vitamin D intake, obesity, fat mass, nutritionist

## Abstract

Prader–Willi syndrome (PWS) is a genetic disorder characterized by hyperphagia with progressive, severe obesity, and an increased risk of obesity-related comorbidities in adult life. Although low dietary vitamin D intake and low 25-hydroxy vitamin D (25OHD) levels are commonly reported in PWS in the context of bone metabolism, the association of low 25OHD levels with fat mass has not been extensively evaluated in PWS adults. The aims of this study were to investigate the following in PWS adults: (1) 25OHD levels and the dietary vitamin D intake; (2) associations among 25OHD levels with anthropometric measurements and fat mass; (3) specific cut-off values for body mass index (BMI) and fat mass predictive of the 25OHD levels. In this cross-sectional, single-center study we enrolled 30 participants, 15 PWS adults (age 19–41 years and 40% males) and 15 control subjects matched by age, sex, and BMI from the same geographical area (latitude 40° 49’ N; elevation 17 m). Fat mass was assessed using a bioelectrical impedance analysis (BIA) phase-sensitive system. The 25OHD levels were determined by a direct competitive chemiluminescence immunoassay. Dietary vitamin D intake data was collected by three-day food records. The 25OHD levels in the PWS adults were constantly lower across all categories of BMI and fat mass compared with their obese counterpart. The 25OHD levels were negatively associated with BMI (*p* = 0.04), waist circumference (*p* = 0.03), fat mass (*p* = 0.04), and dietary vitamin D intake (*p* < 0.001). During multiple regression analysis, dietary vitamin D intake was entered at the first step (*p* < 0.001), thus explaining 84% of 25OHD level variability. The threshold values of BMI and fat mass predicting the lowest decrease in the 25OHD levels were found at BMI ≥ 42 kg/m^2^ (*p* = 0.01) and fat mass ≥ 42 Kg (*p* = 0.003). In conclusion, our data indicate that: (i) 25OHD levels and dietary vitamin D intake were lower in PWS adults than in the control, independent of body fat differences; (ii) 25OHD levels were inversely associated with BMI, waist circumference, and fat mass, but low dietary vitamin D intake was the major determinant of low vitamin D status in these patients; (iii) sample-specific cut-off values of BMI and fat mass might help to predict risks of the lowest 25OHD level decreases in PWS adults. The presence of trained nutritionists in the integrated care teams of PWS adults is strongly suggested in order to provide an accurate nutritional assessment and tailored vitamin D supplementations.

## 1. Introduction

Low vitamin D status is frequently observed in obesity [1,2]. Among the possible mechanisms linking low vitamin D status with obesity, a current explanation is the increased sequestration of 25-hydroxy vitamin D (25OHD) and the volumetric dilution of ingested or cutaneously synthesized vitamin D_3_ in excess adipose tissue [1]. In addition, both dietary vitamin D intake and use of vitamin D supplementation have been reported as consistently lower among obese subjects compared with their normal-weight counterparts [3].

Prader–Willi syndrome (PWS) is the most common syndromic form of childhood obesity, whose prevalence is approximately 1 in 10.000 to 30.000 births. PWS patients present a neurodevelopmental disorder due to an alteration of active paternally expressed genes from the chromosome 15q11-q13 region [4]. The syndrome is characterized by neonatal hypotonia, distinctive facial features, skeletal disorders, developmental and cognitive delay, and behavioral alterations (such as obsessive-compulsive characteristics and hyperphagia starting in early childhood), with the gradual development of morbid obesity (which is associated with endocrine abnormalities such as growth hormone deficiency and short stature) and hypogonadotropic hypogonadism [5]. All these clinical conditions interfere with the quality of life of these patients in adulthood [4]. Despite improved diagnoses and management of PWS and a relative preservation of insulin sensitivity in PWS youth, PWS adults usually tend to develop severe obesity-related comorbidities during their lifetimes [6]. In both PWS youths [7,8] and adults [9], low dietary vitamin D intake and low vitamin D status are commonly reported in the context of bone metabolism. To the best of our knowledge, the association of low vitamin D status with fat mass has not been extensively evaluated in PWS adults; however, vitamin D deficiency has been associated with obesity per se, along with the vast majority of obesity-related comorbidities and increased cardiovascular risk [10,11,12,13,14,15,16].

Body composition assessment—fat mass in particular—is crucial evaluating of the state of an individual’s health, because high values may contribute at a higher risk of several diseases [17]. Dual energy X-ray absorptiometry (DXA) is the current gold standard method for assessing body composition; however, it is quite expensive and is present only in few centers. Bioelectrical impedance analysis (BIA) represents a widely employed method used to evaluate body composition based on the use of prediction equations obtained by cross-validating BIA against DXA [18]. BIA devices have been used in many studies in populations with obesity, including cases of PWS [19,20,21,22].

Despite some controversy, a large body of evidence indicates that PWS patients are characterized by a unique altered body composition consisting of a fat mass higher than expected for the degree of weight excess and higher than that of their obese counterparts with comparable BMIs [21,23,24,25,26,27,28,29,30,31]. This is associated with a decrease in muscle mass by 25–37% [27], largely contributing to the burden of disease.

In this context, PWS could represents a useful model for determining the relative contributions of diet, obesity, and fat mass on vitamin D status.

The aims of this study were to determine the following in PWS adults: (1) 25OHD levels and dietary vitamin D intake; (2) associations among 25OHD levels and anthropometric and BIA measurements; (3) specific cut-off values for BMI and fat mass that are predictive of 25OHD levels.

## 2. Material and Methods

### 2.1. Design and Setting

This was a cross-sectional, observational, single-center study performed at Department of Clinical Medicine and Surgery, Unit of Endocrinology, University Federico II, Naples (Italy), from October 2016 to January 2020. The study was been carried out taking into account the Code of Ethics of the World Medical Association (Declaration of Helsinki) for experiments involving humans. The protocol was formally approved by the Ethical Committee of the University of Naples Federico II Medical School (n. 173/16). Every enrolled subject provided an informed consent after a thorough explanation of the protocol.

### 2.2. Population Study

Recruitment strategies included 15 patients with genetically confirmed diagnoses of PWS (via positive methylation testing) who all attended the same department of the Outpatient Clinic of the Unit of Endocrinology. Fifteen Caucasian subjects with obesity were chosen as controls among hospital volunteers and employees from the same geographical area around Naples metropolitan area, Italy. In order to increase the homogeneity of the subject samples we included adults of both genders with the following criteria of exclusion:

- type 2 diabetes mellitus;
- chronic diseases that could interfere with fluid homeostasis, such as liver or chronic renal diseases, cancer, and acute or chronic inflammatory diseases;
- altered levels of serum creatinine, serum calcium, or albumin;
- current therapy with calcium, osteoporosis therapies, and medications that may affect vitamin D absorption or metabolism including anti-inflammatory drugs, sex hormone therapy, statins and other hypolipidemic agents, and medications to reduce body weight;
- pacemakers or defibrillators that could potentially interfere with BIA assessment.

Study participants were asked to complete an interview with the principal investigator about demographic data, medical history and current medical status, vitamin D supplementation, drugs, treatment duration, and smoking habits. In both groups, all female subjects were assessed during the follicular phase of their menstrual cycle. They did not report being pregnant or lactating. All participants were enrolled in the autumn-winter seasons (October to March) only, and all were from the same geographical area around Naples metropolitan area, Italy (latitude 40° 49′ N; elevation 17 m). Therefore, all subjects had similar sun exposure. In addition, none of the participants reported any sun exposure at the time of enrollment, was engaged in leisure time physical activity, or used vitamin D supplementation. All PWS patients in our cohort were previously treated with recombinant human growth hormone (rhGH) treatment during childhood. The treatment was stopped at least two years before starting the study.

### 2.3. Power Size Justification

The power sample was calculated by the differences of means ± standard deviation (SD) of 25OHD levels in PSW adults and the control group (9 ± 3 vs 14 ± 4 ng/mL, respectively). Considering the number of cases required for each group was nine out of 15, with a type I (alpha) error of 0.05 (85%) and a type II (beta) of 0.05, the calculated power size was 95%. The calculation of sample size and power were performed while using a Sample Size Calculator Clinical Calc (https://clincalc.com/stats/samplesize.aspx).

### 2.4. Anthropometric Measurements

Measurements were performed in the morning between 8:00 a.m. and 12:00 p.m. after an overnight fast. The anthropometric measurements were performed following standard criteria from the same nutritionist according to the International Society for the Advancement of Kinanthropometry (ISAK 2006). The subjects were recommended to dress in light clothes and remove shoes during the assessment, as previously reported [32,33,34,35].

BMI (weight (kg) divided by height squared (m^2^), kg/m^2^) was calculated after measuring weight and height. A wall-mounted stadiometer (Seca 711; Seca, Hamburg, Germany) was used to measure height while a calibrated balance beam scale (Seca 711; Seca, Hamburg, Germany) was used to assess weight. The degree of obesity was established according to the World Health Organization (WHO)’s criteria: BMI: 30–34.9 kg/m^2^, grade I obesity; BMI: 35–39.9 kg/m^2^, grade II obesity; BMI ≥40 kg/m^2^, grade III obesity [36]. A single nutritionist measured waist circumference using a non-stretchable tailor measuring tape placed around the bare abdomen just above the hip bone and parallel to the floor. Patients and the control group were asked to exhale, and measurements were taken to the nearest centimeter at the midpoint between the bottom of the rib cage and above the top of the iliac crest during minimal respiration [37].

### 2.5. Bioelectrical Impedance Analysis

As previously reported [38,39,40,41], a BIA phase-sensitive system (an 800-µA current at a single-frequency of 50 kHz BIA 101 RJL, Akern Bioresearch, Florence, Italy) was used by experienced observers to assess body composition [42]. We performed the exam as suggested by the European Society of Parental and Enteral Nutrition (ESPEN) [43]. Electrodes were placed on the hand and ipsilateral foot according to Kushner [44]. The fat mass was estimated using the prediction equation for adult female PWS patients developed by Bedogni et al. [21] and for male patients by Gray et al. [45], which has also previously been reported by Lazzer et al. [46].

A single nutritionist performed all measurements under strictly standardized conditions, using the same BIA device in order to avoid interobserver and interdevice variability. The BIA device was routinely checked with resistors and capacitors of known values. Reliability according the same observer were <1.6% for resistance and <1.8% for reactance for within-day measurements, and <2% for resistance and <2% for reactance for between-day measurements. The coefficients of variation (CVs) of repeated measurements of resistance and reactance at 50 kHz were assessed in five individuals according the same observer: CVs were 1% for resistance and 1% for reactance.

### 2.6. Dietary Records

All participants or their parents completed a three-day food record as accurately and thoroughly as possible using the serving sizes [8,9]. As we have already fully reported in previous studies [47,48,49,50]. A photographic food atlas (≈1000 photographs) of known portion sizes was used to quantify foods and drinks [51]. Data were stored and processed later by a single nutritionist, using a specific software (Terapia Alimentare Dietosystem DS-Medica (http://www.dsmedica.info)) that also calculates dietary vitamin D intake. Taking into account possible underreporting, the dietary vitamin D intake is expressed as amount per 1000 kcal of total energy intake. RDA for vitamin D (≥15 µg) is given by Italy’s National Recommended Energy and Nutrient Intake Levels (LARN) [52].

### 2.7. Assay Methods

Samples were collected in the morning between 8:00 a.m. and 12:00 p.m. after an overnight fast of at least 8 h and stored at −80 °C until being processed. The serum levels of 25OHD were quantified by a direct competitive chemiluminescence immunoassay (CLIA) (Liaison, DiaSorin, Saluggia, Italy), with a specificity of 100% for 25OHD. The analytical measurement range of detection was 4–150 ng/mL, whereas the intra-assay CVs were 5%, 3%, and 5%, and the inter-assay CVs were 10%, 5%, and 8% for low, medium, and high points of the standard curve, respectively (as previously reported in [53,54,55,56]). The vitamin D deficiency was defined as 25OHD levels < 20 ng/mL (50 nmol/L), vitamin D insufficiency as 25OHD levels between 21 and 29 ng/mL (from 53 to 73 nmol/L) and vitamin D sufficiency as 25OHD levels ≥30 ng/mL (75 nmol/L) [57].

### 2.8. Statistical Analysis

The data distribution was evaluated by a Kolmogorov–Smirnov test and the abnormal data were normalized by a logarithm. Skewed variables were back-transformed for presentation in tables and figures. Results are expressed as mean ± SD and categorical variables are expressed as a percentage.

Differences between PSW adults and the control group were analyzed by a Student’s independent *t*-test; the chi square (χ^2^) test was used to determine the significance of differences in the frequency distribution of BMI categories.

The correlations among 25OHD levels, age, BMI, waist circumference, and fat mass were assessed with Pearson *r* correlation coefficients.

A multiple linear regression analysis model (stepwise method) expressed as R^2^, Beta (β), and *t*, with 25OHD levels as the dependent variable, was used to estimate the predictive values of variables associated with 25OHD levels in the univariate analyses (BMI, waist circumference, fat mass, and dietary vitamin D intake).

Receiver operator characteristic (ROC) curve analysis was carried out in order to identify sensitivity and specificity, area under the curve (AUC), and confidence interval (CI), as well as cut-off values of BMI and fat mass in detecting the lowest decrease in 25OHD levels. AUC tests were also performed to analyze ROCs, and we entered 0.88 for the AUC ROC and 0.5 for the null hypothesis values. An Alfa level of 0.05 (type 1 error) and a β level of 0.2 (type II error) were used as the cut-off values for statistical significance. Variables with a variance inflation factor (VIF) > 10 were excluded in order to avoid multicollinearity. Values ≤ 5% were considered statistically significant. Data were collected and analyzed using the MedCalc package (Version 12.3.0 1993- 2012 -Mariakerke, Belgium).

## 3. Results

The study population consisted of 30 participants: 15 PWS adults and 15 controls matched by age, sex, and BMI. Age, anthropometric characteristics, and dietary vitamin D intake of the study population are summarized in Table 1. There were no significant differences in waist circumference or fat mass between PWS adults and controls. Nearly all participants demonstrated a dietary vitamin D intake lower than RDA, but in PWS adults the dietary vitamin D intake was significantly lower than the control (*p* = 0.01).

**Figure 1:**
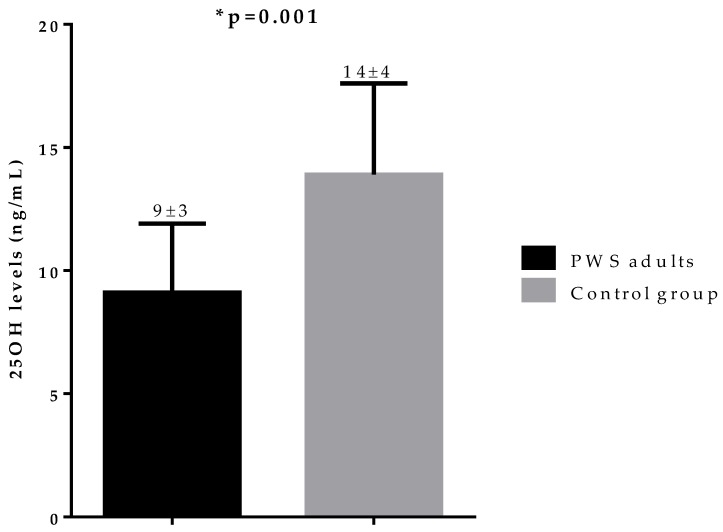
Difference in 25OHD levels in PWS adults and the control group. PWS adults showed lower 25OHD levels compared to matched controls (9 ± 3 vs 14 ± 4 ng/mL, *p* = 0.001). * A *p* value < 0.05 means a significant difference. ***PWS,*** Prader–Willi syndrome.

Figure 1 shows the 25OHD levels in PWS adults and the control group. All study participants presented with vitamin D deficiency, but the 25OHD levels in PWS adults were lower than in the control group.

**Figure 2:**
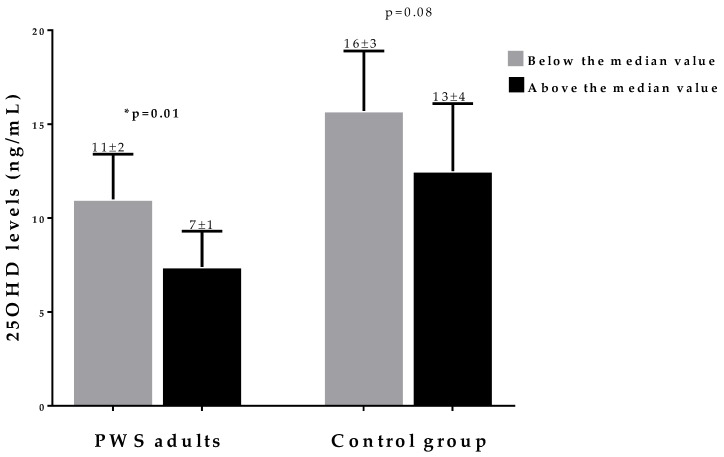
Differences in 25OHD levels in PWS adults and in the control group within the BMI categories (above and below the median values). BMI mean values were 42 and 44 Kg/m^2^ in PWS adults and the control group, respectively. The 25OHD levels were lower in the BMI category above the median value in PWS adults only (11 ± 2 vs 7 ± 1 ng/mL, *p* = 0.01). * A *p* value < 0.05 means a significant difference. ***PWS,*** Prader–Willi syndrome.

**Figure 3:**
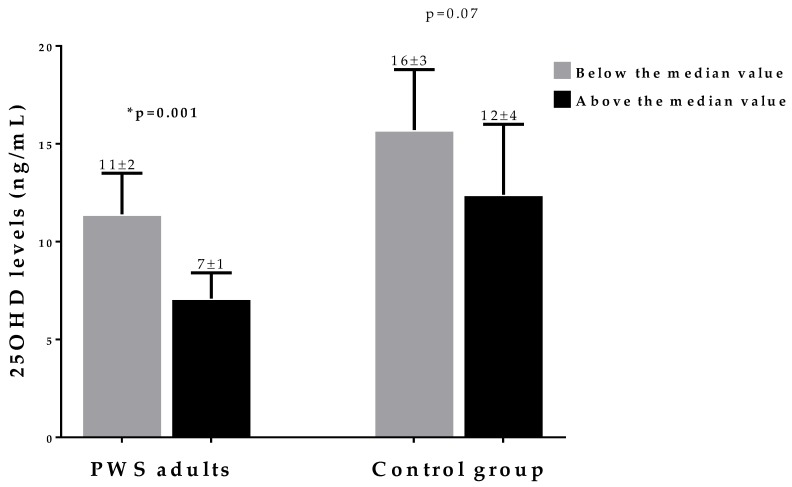
Differences in 25OHD levels in PWS adults and in the control group within fat mass categories (above and below the median values). Fat mass median values were 46 and 58 Kg in PWS adults and the control group, respectively. The 25OHD levels were lower in the fat mass category above the median values in PWS adults only (11 ± 2 vs 7 ± 1; *p* = 0.001). * A *p* value < 0.05 means a significant difference. ***PWS,*** Prader–Willi syndrome.

Differences in the 25OHD levels within BMI and fat mass categories (above and below the median value) between PWS adults and the control group are reported in Figure 2 and Figure 3, respectively. BMI mean values were 42 and 44 Kg/m^2^, respectively, and fat mass mean values were 46 and 58 Kg, respectively. While in the control there were no differences in 25OHD levels between subjects within each BMI and fat mass category, 25OHD levels were lower in PWS adults with a BMI and fat mass above their median values, as compared with those below the median values (*p* = 0.01 and *p* = 0.001, respectively).

**Figure 4:**
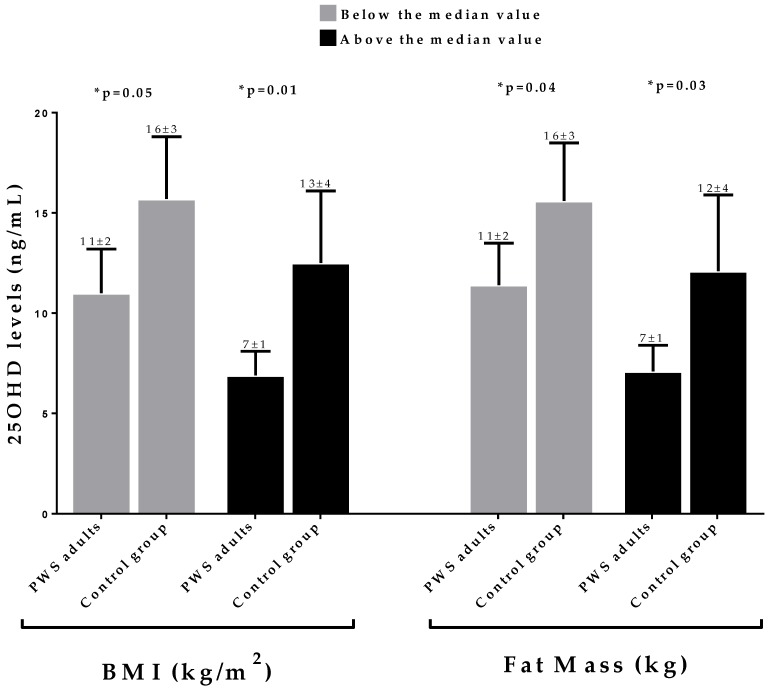
The differences in 25OHD levels between PWS adults and the control group for each BMI and fat mass category (above and below the median values). In each BMI and fat mass category the 25OHD levels were significantly lower in PWS adults than in the control group. * A *p* value < 0.05 means a significant difference. ***PWS,*** Prader–Willi syndrome; ***BMI,*** Body Mass index.

However, considering each BMI and fat mass category (below and above the median values), the 25OHD levels were significantly lower in PWS adults than in the control (Figure 4).

### Correlation Analysis

Correlations of 25OHD levels with age, anthropometric measurements, fat mass, and dietary vitamin D intake are reported in Table 2. Apart from age, the 25OHD levels showed significant associations with all parameters included in the analysis.

In the multiple regression analysis using BMI, waist circumference, fat mass, and dietary vitamin D intake as independent variables and 25OHD levels as the dependent variable, dietary vitamin D intake was entered at the first step (*p* < 0.001), explaining 84% of 25OHD level variability. Results are shown in Table 3.

**Figure 5:**
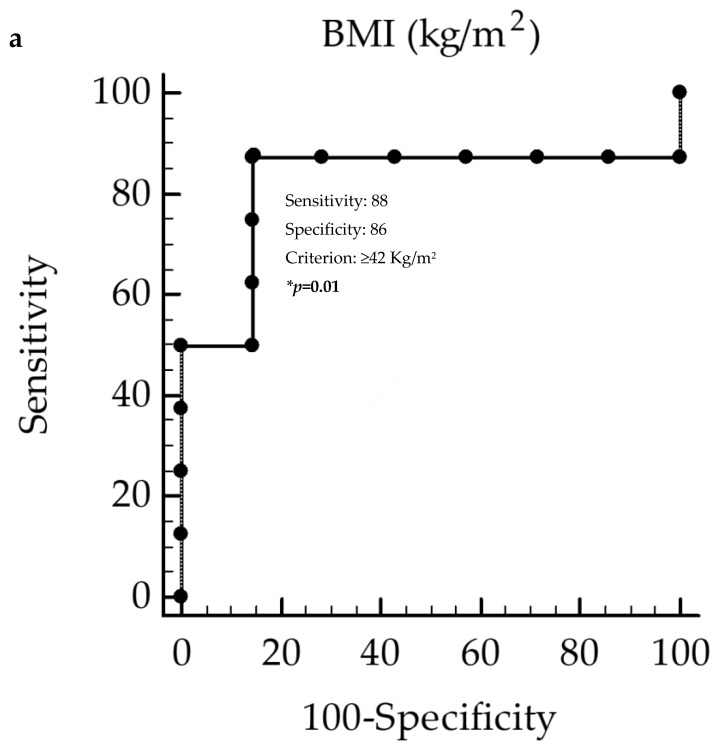

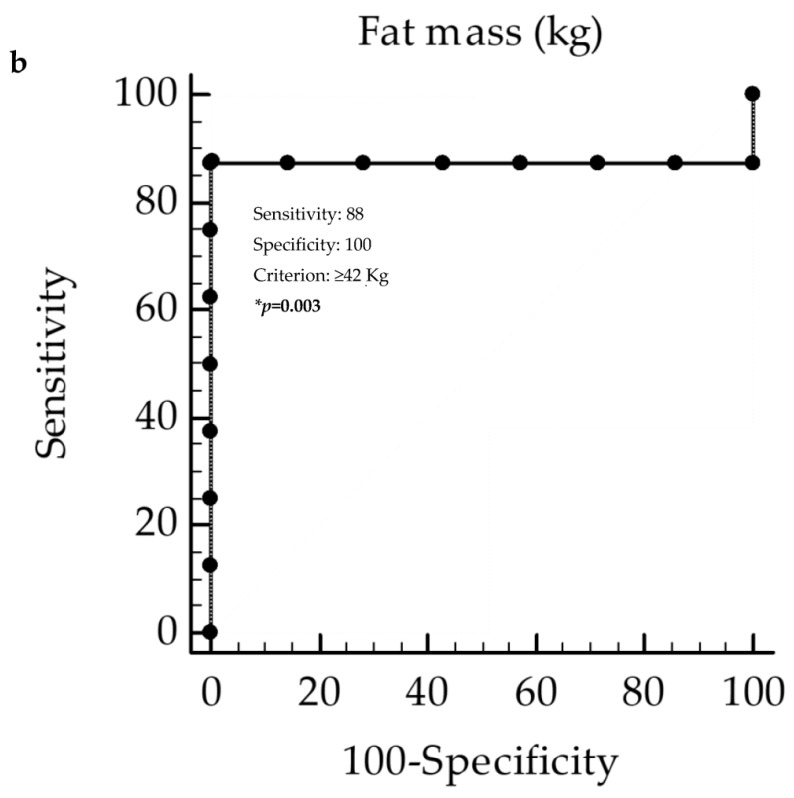
ROC for predictive values of BMI (**a**) and fat mass (**b**) in detecting the lowest decrease in 25OHD levels. * A *p* value in bold type denotes a significant difference (*p* < 0.05). ***ROC,*** Receiver Operator Characteristic; ***BMI,*** Body Mass Index.

In the ROC analysis, the threshold values of BMI and fat mass predicted the lowest decreases in 25OHD levels (below the median 9 ng/mL) to be at BMI ≥ 42 kg/m^2^ (*p* = 0.01, AUC 0.81, standard error 0.13, 95% CI 0.6 to 0.9; Figure 5a) and fat mass ≥ 41 Kg (*p* = 0.003, AUC 0.88, standard error 0.12, 95% CI 0.6 to 0.9; Figure 5b).

## 4. Discussion

This cross-sectional, single-center study found that PWS adults had lower 25OHD levels than age-, sex-, and BMI-matched subjects, and that this was associated with a lower dietary vitamin D intake. The low vitamin D status in these patients was independent of waist circumference and fat mass, as we did not observe any significant differences in these variables between PWS adults and controls. Nevertheless, while in the control group there were no differences in 25OHD levels within each BMI and fat mass category, in PWS adults the 25OHD levels were significantly reduced among subjects with a higher BMI and fat mass. In addition, 25OHD levels were constantly lower across all categories of BMI and fat mass in PWS adults compared with their obese counterpart. Of interest, BMI ≥ 42 kg/m^2^ and fat mass ≥ 42 Kg, respectively, resulted the most sensitive and specific cut-off points to predict values of the lowest decreases in 25OHD levels.

As reported above, we did not observe any significant differences in fat mass between PWS adults and the control group. Although the difference in fat mass between PWS adults and their obese counterpart with a comparable BMI has gained considerable support from some studies (as reported above [23,24,25,26,27,28,29,30,31]), this evidence has not been steadily reported by other DXA studies of either PWS children [58] or adults [59]. In particular, McAlister et al. (2018) reported a percentage of body fat in a cohort of 21 youth PWS patients that was similar to a control group of non-syndromic individuals with obesity, which they associated with a more favorable cardio-metabolic profile [58]; however, all youth PWS participants in this study were receiving a rhGH treatment. Similarly, Viardot et al. (2018) found no significant differences in fat mass between a cohort of 11 PWS adults and a weight-matched obese group, but none of the PWS adults in this study had previously received rhGH treatment [59]. In our study, it is conceivable that a previous treatment with rhGH in childhood could have accounted for the lack of differences in fat mass between PWS adults and controls.

Evidence of low 25OHD levels and dietary vitamin D intake in PWS has been shown in two recent studies on PWS youth [7,8]. In particular, Fintini et al. (2018) evaluated vitamin D status in a series of 52 Italian children and adolescents with PWS, reporting that vitamin D deficiency in these patients was comparable to that observed in 110 controls matched for age, sex, and BMI [7]. In addition, Mackenzie et al. (2018) examined dietary intake and quality in a cohort of 23 Canadian youths with PWS who were compared to a control group. It was found that more than half of the youths with PWS were at risk of inadequate vitamin D and calcium intake, despite similar nutrient intakes and only a trend towards higher BMI z-scores in the PWS group [8]. However, body composition was not evaluated in the latter study, while in the Italian study the authors reported a negative correlation between 25OHD levels and fat mass in both groups, despite the youth PWS patients presenting a higher fat mass percentage than the controls. Similarly, Woods et al. (2018) confirmed that there is inadequate dietary vitamin D intake in adulthood PWS, as they showed through a study of 19 PWS adults, none of whom met vitamin D recommendations from diet alone (25OHD levels were not provided) [9]. Of interest, the authors of this study showed that their PWS adults had lower body fat percentages than the percentages reported in most studies, especially in PWS adults of a younger age, likely due to earlier diagnoses and interventions in diet, exercise, and daily routines, as well as through use of rhGH replacement therapy. Finally, Brunetti et al. (2018) investigated bone metabolism in 12 PWS children and 14 PWS adults and reported that 25OHD levels in both groups were significantly reduced compared with the controls, although 25OHD mean values were >20 ng/mL [60]. In our study, dietary vitamin D intake was significantly lower in PWS adults than in the control group, and, consequently, 25OHD levels were lower among PWS adults across all categories of BMI and fat mass compared with their obese counterpart.

The novelty of this study is to provide information on vitamin D status in PWS adults and on what the most important determinants of vitamin D status are in this population specifically (dietary vitamin D intake or fat mass). Our findings indicate that PWS adults might be at a higher risk of vitamin D inadequacy than their obese counterparts, and that vitamin D status should be included in endocrine and metabolic assessments of PWS adults. In addition, the results of the present study do not lend support to the thesis that the extent of adipose tissue is a main determinant of low vitamin D status in PWS adults, while it is evidenced that the dietary vitamin D intake is the major determinant of 25OHD levels. As the estimation of dietary vitamin D intake requires an accurate dietary assessment performed by a trained nutritionist (who are not always present in a multidisciplinary care team facing PWS adults), we have provided sample-specific cut-off points of BMI and fat mass, which are variables that are easy to obtain in a clinical setting and can predict low vitamin D status in PWS adults.

The low vitamin D status in PWS adults raises some considerations. The common treatments to counteracting the pathological characteristics of PWS—which include restricted caloric intake, increased physical activity, and rhGH therapy—are making it possible for PWS adults to live longer. Nevertheless, obesity-related comorbidities still remain the major contributors to morbidity and mortality in these patients, with the mortality rate reported up to 7% per year for those over 30 years of age [9]. Reduced intake of food containing vitamin D, less sunlight exposure, and scarce or absent physical activity (with preference given to indoor leisure activities) are all conditions that contribute to an increased risk of vitamin D deficiency in PWS adults. Taking into account the growing evidence of bidirectional, albeit debatable, relationships between vitamin D, obesity, and several chronic diseases [1,2,10,11,12,13,61,62], the importance of a sufficient vitamin D status could be of strategic relevance to managing the metabolic consequences of PWS in adults, in addition to the prevention and the treatment of bone alterations in these patients. Thus, trained nutritionists should be an integral part of any multidisciplinary care team, in order to ensure the adequate intake and/or supplements of micronutrients such as vitamin D.

We are aware that there are some limitations in the current study. First, the cross-sectional nature of this study did not allow any statements on the causal relationships among 25OHD levels, dietary vitamin D intake, and fat mass in PWS adults. Second, the sample size was relatively small. Nevertheless, we have calculated the sample size using 95% power. The number of cases required in each group was nine, while we used 15 patients for each group. In addition, the control group was matched for age, sex and BMI, making it possible to compare these variables independently across subjects. In addition, as the study was based on a single clinical center, all study participants lived in the same geographical area, with the same effect of latitude on 25OHD levels and, likely, similar nutrient availability and food consumption patterns, which allowed us to increase the homogeneity of the study sample. Third, the proposed cut-off points of BMI and fat mass for identifying the lowest decreases in 25OHD levels should be viewed with caution until the results of clinical studies in larger population samples have been made available, and appropriate cross-validation can be performed. Finally, although DXA is considered the ‘gold standard’ for measurement of body composition, BIA was used, as it has been reported to be a validated method, providing a useful alternative for measuring body fat in clinical practice (with a high agreement with DXA results) as well as among patients with severe obesity [63], and has been largely used in other studies in PWS [19,20,21,22]. To avoid inter-operator variability, only one expert nutritionist evaluated the anthropometric measures, administered the three-day food records, and assessed, executed, and interpreted the BIA measurements.

## 5. Conclusions

In summary, our data indicate the following in PWS adults: (i) 25OHD levels were lower compared with the control group, independent of body fat; (ii) 25OHD levels were inversely associated with BMI, waist circumference, and fat mass, and low dietary vitamin D intake was the major determinant of low vitamin D status in these patients; (iii) sample-specific cut-off values of BMI and fat mass might help to predict the lowest decreases in 25OHD levels in PWS adults. Hence, we suggest that the evaluation of vitamin D status, in association with accurate nutritional assessment, should be routinely performed in PWS adults by an integrated care team including trained nutritionists, to accurately assess vitamin D status and provide a tailored vitamin D supplementation.

## Figures and Tables

**Table 1:** Gender, age, anthropometric characteristics, fat mass, and dietary vitamin D intake of the study population.

Parameters	PWS Adults Mean ± SD or Number (%) n = 15	Controls Mean ± SD or Number (%) n = 15	*p*-Value
Gender (M/F)	6/9 (40/60%)	6/9 (40/60%)	χ^2^ = 0.14, *p* = 0.71
Age (years)	28 ± 7	30 ± 7	0.66
BMI (kg/m^2^)	44 ± 11	44 ± 9	0.21
Grade I obesity	3, 20%	2, 13%	χ^2^ = 0.34, *p* = 0.84
Grade II obesity	3, 20%	4, 27%	
Grade III obesity	9, 60%	9, 60%	
Waist Circumference (cm)	123 ± 28	113 ± 19	0.25
Males	136 ± 33	124 ± 16	0.49
Females	115 ± 21	105 ± 17	0.37
Fat Mass (kg)	50 ± 24	62 ± 23	0.26
Males	54 ± 34	73 ± 24	0.36
Females	47 ± 18	54 ± 19	0.51
Dietary vitamin D intake (μg/1.000 kcal)	4 ± 1	5 ± 1	**0.01**

A *p* value in bold type denotes a significant difference (*p* < 0.05). ***PWS,*** Prader–Willi syndrome; ***SD,*** standard deviation; ***M,*** Males; ***F,*** Females.

**Table 2:** Correlations among 25OHD levels with age, anthropometric measurements, fat mass, and dietary vitamin D intake.

Parameters.	n = 15	
	r	*p*-Value
Age (years)	0.12	0.68
BMI (kg/m^2^)	−0.52	**0.04**
Waist circumference (cm)	−0.56	**0.03**
Fat mass (kg)	−0.52	**0.04**
Dietary vitamin D intake (μg/1.000 kcal)	0.91	**<0.001**

A *p* value in bold type denotes a significant difference (*p* < 0.05). ***BMI***, Body Mass Index.

**Table 3:** Multiple regression analysis models (stepwise method) with 25OHD levels as the dependent variable to estimate the predictive values of BMI, waist circumference, fat mass, and dietary vitamin D intake.

Parameters.	Multiple Regression Analysis			
	R^2^	β	t	*p*-Value
**Dietary Vitamin D Intake (μg/1.000 kcal)**	0.84	0.92	8.2	**<0.001**
*Excluded variables: BMI, waist circumference, and fat mass*				

A *p* value in bold type denotes a significant difference (*p* < 0.05). ***BMI,*** Body Mass Index.

## Abbreviations

PWS	Prader–Willi syndrome; 25OHD, 25-hydroxy vitamin D
BMI	body mass index
RDA	recommended dietary allowance
DXA	dual energy X-ray absorptiometry
BIA	bioelectrical impedance analysis
rhGH	recombinant human growth hormone
SD	standard deviation
CVs	coefficients of variation
ROC	receiver operator characteristic
AUC	area under the curve
CI	confidence interval
